# *QuickStats:* Prevalence* of Untreated Dental Caries^^†^^ in Permanent Teeth Among Children and Adolescents Aged 6–19 Years, by Age Group — National Health and Nutrition Examination Survey, United States, 2011–2014

**DOI:** 10.15585/mmwr.mm6601a11

**Published:** 2017-01-13

**Figure:**
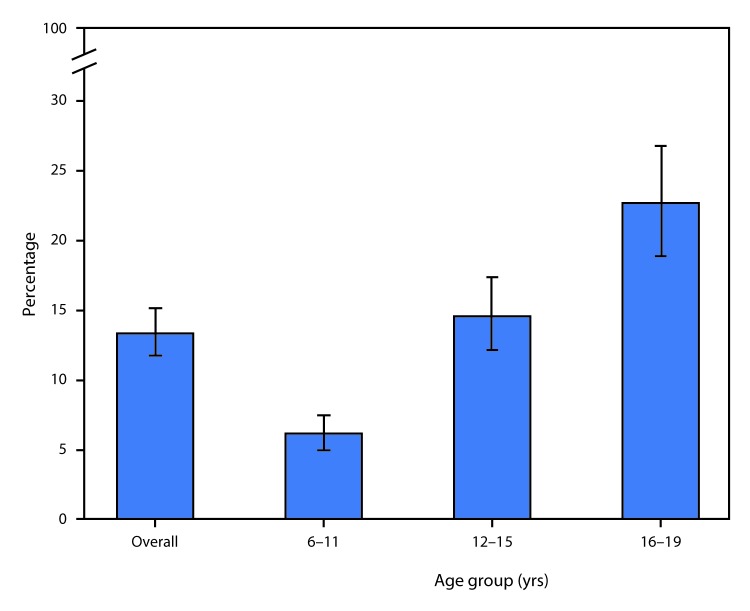
During 2011–2014, 13.3% of children and adolescents aged 6–19 years had untreated dental caries in their permanent teeth. The percentage of children and adolescents with untreated dental caries increased with age: 6.1% among those aged 6–11 years, 14.5% among those aged 12–15 years, and 22.6% among those aged 16–19 years.

**Source: **National Health and Nutrition Examination Survey data. Hyattsville, MD: US Department of Health and Human Services, CDC, National Center for Health Statistics; 2011–2014. https://www.cdc.gov/nchs/nhanes.htm.

